# Prognostic significance of survival-associated alternative splicing events in gastric cancer

**DOI:** 10.18632/aging.104013

**Published:** 2020-11-07

**Authors:** Shichao Zhang, Zuquan Hu, Yingwu Lan, Jinhua Long, Yun Wang, Xiaowen Chen, Xiaofeng Xu, Zhu Zeng, Yan Ouyang

**Affiliations:** 1 Immune Cells and Antibody Engineering Research Center of Guizhou Province, Key Laboratory of Biology and Medical Engineering, School of Biology and Engineering/School of Basic Medical Sciences, Guizhou Medical University, Guiyang 550025, Guizhou, P.R. China; 2 Key Laboratory of Environmental Pollution Monitoring and Disease Control, Ministry of Education of China, Guiyang 550025, Guizhou, P.R. China; 3 Affiliated Tumor Hospital, Guizhou Medical University, Guiyang 550025, Guizhou, P.R. China; 4 The Clinical Laboratory of Beijing Tongren Hospital, Capital Medical University, Beijing 100730, P.R. China

**Keywords:** gastric cancer, alternative splicing events, prognostic signature, survival, splicing factors

## Abstract

Alternative splicing events are a major source of transcript and protein diversity in eukaryotes. Aberrant alternative splicing events have been increasingly reported in various cancers, including gastric cancer. To further explore the prognostic significance of alternative splicing events in gastric cancer patients, a comprehensive and systematic investigation was conducted by integrating alternative splicing event data and clinical information. Univariate Cox regression analysis identified 1383 alternative splicing events to be significantly associated with the overall survival of gastric cancer patients. Then, least absolute shrinkage and selection operator (LASSO) and multivariate Cox analyses were performed for the development of prognostic signatures. The final prognostic signature based on all seven types of alternative splicing events can act as an independent prognostic indicator after multivariate adjustment of several clinical parameters. Furthermore, the correlation and function analysis identified *CELF2*, *BAG2*, *RBFOX2*, *PTBP2* and *QKI* as hub splicing factors, and the focal adhesion signaling pathway was most significantly correlated with survival-associated alternative splicing events. The results of this study may establish a foundation for further research investigating the underlying mechanism of alternative splicing events in the progression of gastric cancer.

## INTRODUCTION

Alternative splicing (AS) can edit a single pre-mRNA molecule and produce diverse mature mRNAs in eukaryotic organisms. These transcript variants can subsequently generate proteins with different structures and biological functions. Therefore, AS is an important mechanism for posttranscriptional regulation of gene expression and plays a vital role in the diversification of both the transcriptome and the encoded proteome [1]. Generally, there are seven main patterns of AS events, such as exon skip (ES), retained intron (RI), alternate donor site (AD), alternate acceptor site (AA), alternate promoter (AP), alternate terminator (AT) and mutually exclusive exons (ME) [2, 3]. Recent high-throughput sequencing studies indicate that >95% of genes undergo AS and generate at least two alternative pre-mRNA isoforms [1, 4]. Aberrant AS events may lead to multiple pathological processes, especially cancer initiation, progression, metastasis and resistance to therapy [5–8]. AS events could be developed as diagnostic or prognostic biomarkers, as well as for exploiting therapeutic targets in cancer patients [2].

Gastric cancer (GC) is one of the most common malignant tumors that originate from the gastric mucosal epithelium. It has been reported that GC has the second highest incidence among various cancers in China and ranks as the third leading cause of cancer-related death worldwide [9, 10]. After the importance of AS events in Epstein-Barr virus-associated GC was investigated [11], a simple prognosis analysis was conducted to assess AS events in stomach adenocarcinoma [12]. However, there is still a lack of clinical references regarding the prognostic value of AS, and the regulatory mechanism governing survival-associated AS events warrants further study. Thus, an in-depth and systematic investigation of survival-associated AS events in GC patients should be conducted to build an independent prognostic signature by integrating seven types of AS events, which may provide suggestions for exploiting personalized treatment strategies and therapeutic targets.

In this study, we illustrated the effects of different AS patterns in a GC cohort using the genome-wide transcriptome approach. RNA-seq data in The Cancer Genome Atlas (TCGA) were used to analyze the incidence of seven AS patterns and explore splicing variant function and survival-associated AS events in GC patients. The potential regulatory mechanisms for survival-related AS events were revealed. More importantly, a final prognostic signature was successfully constructed by combining seven types of AS events, which was demonstrated to be an independent prognostic indicator after multivariate adjustment of clinical parameters.

## RESULTS

### Integrated AS events in the GC cohort

**Figure 1:**
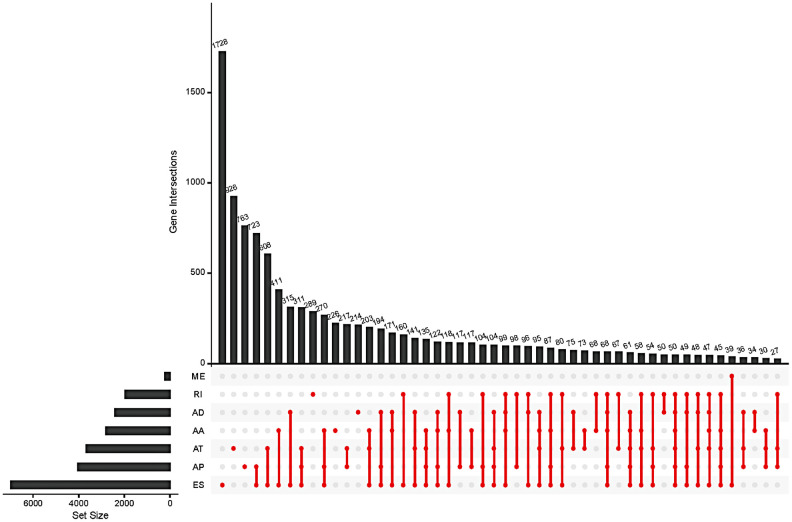
**Summary of total AS occurrence samples in the GC cohort.**

The SpliceSeq package provides a comprehensive, detailed evaluation of seven types of AS events, including AA, AD, AP, AT, ES, ME, and RI. A total of 48141 AS events of 10610 genes were identified in 415 GC patients, showing that a single gene might have more than one type of mRNA splicing event. A single gene may contain up to six types of splicing events. ES was the most frequent splice signature among the seven AS types followed by AT and AP. Specifically, we detected 19121 ESs in 6972 genes, 8390 ATs in 3666 genes, 10004 APs in 4025 genes, 4006 AAs in 2799 genes, 3450 ADs in 2401 genes, 2944 RIs in 1956 genes, and 226 MEs in 219 genes (Figure 1).

### Survival-associated AS events in the GC cohort

**Figure 2:**
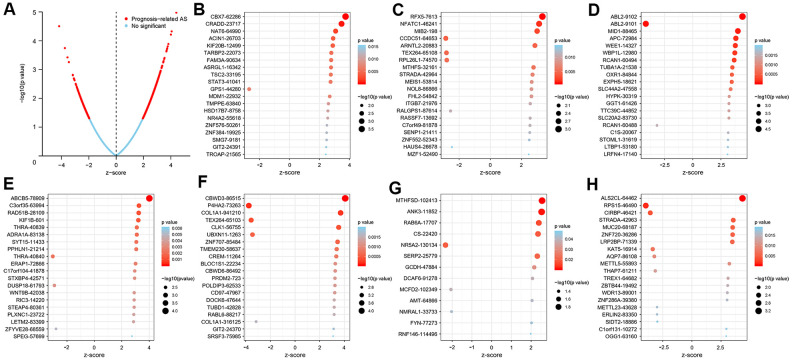
**Top 20 most significant AS events in the GC cohort.** (**A**) The blue dots represent no significant AS events, whereas the red dots represent prognosis-related AS events. The top AS events correlated with survival outcome based on AA (**B**), AD (**C**), AP (**D**), AT (**E**), ES (**F**), ME (**G**), and RI (**H**) events.

**Figure 3:**
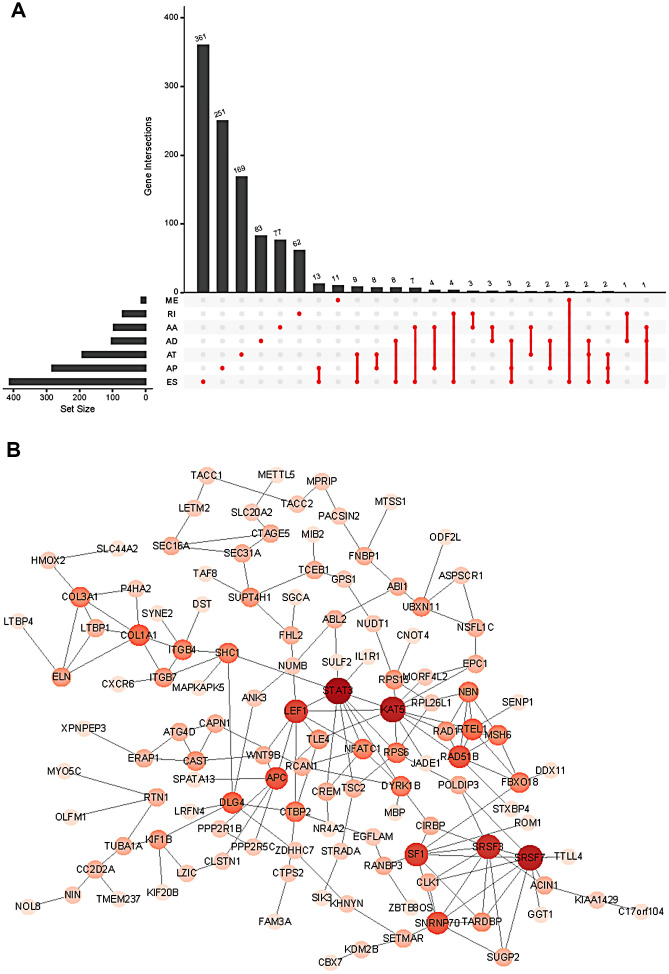
****Summary (**A**) and protein network (**B**) of survival-associated AS events in the GC cohort.

In the survival analysis, 47 patients with an overall survival time of less than 30 days were excluded. The remaining 368 patients were then matched with their corresponding entries in the SpliceSeq database, and 31 cases were excluded once again due to >20% missing AS events. Thus, 337 patients were included in this study and their overall survival status was listed in Supplementary Table 1. To search the prognostic value of AS events in GC patients, a univariate Cox regression analysis was carried out to estimate the influence of each AS event on the overall survival of patients. A total of 1383 AS events were found to be significantly associated with the overall survival of GC patients, including 517 ES events, 354 AP events, 225 AT events, 98 AA events, 104 AD events, 72 RI events, and 13 ME events. The top 20 most significant survival-associated genes in the seven AS events are presented in Figure 2. Notably, most of these AS events were correlated with prognosis, and one gene might have two or more survival-associated splicing events in GC patients. Thus, a subset of overlapping AS events among the seven AS types in GC patients was further analyzed. As illustrated by the UpSet plot diagram in Figure 3A, one gene might undergo two or three types of AS events that were significantly associated with patient survival.

To further explore the functional relationship among these survival-associated AS events, Reactome was used to plot the protein interaction networks. Figure 3B shows the hub genes with survival-associated AS events, such as *STAT3*, *SRSF7*, *KAT5*, *SRSF3*, *SF1*, *LEF1*, *APC*, *RAD51B*, SNRNP70, *COL1A1* and *DLG4*.

### Prognostic predictors for GC patients

**Figure 4:**
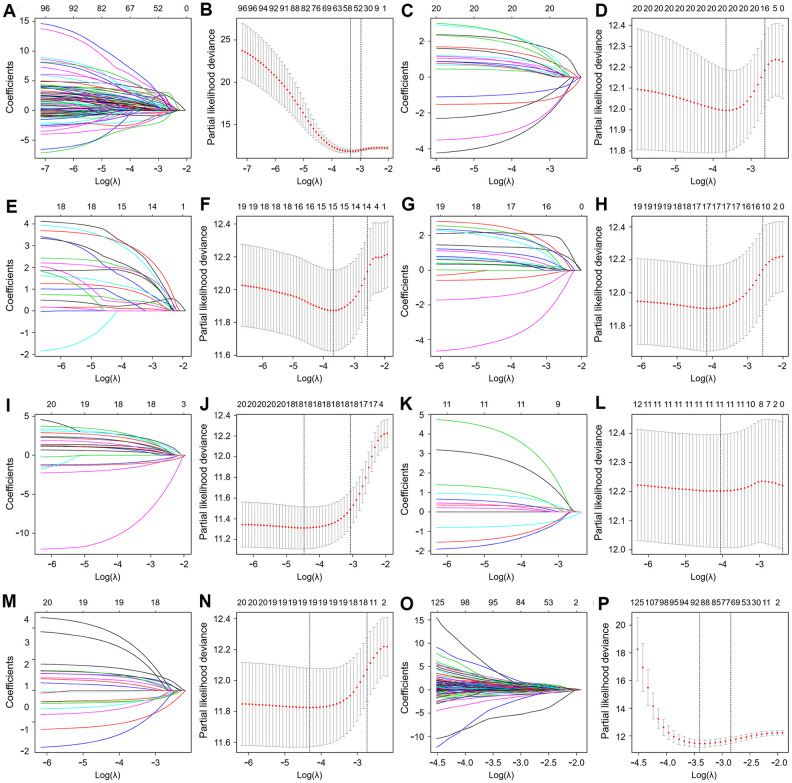
**Least absolute shrinkage and selection operator (LASSO) coefficient profiles of the AS events.** The coefficient profiles of AA (**A**), AD (**C**), AP (**E**), AT (**G**), ES (**I**), ME (**K**) and RI (**M**) events. The partial likelihood deviance of AA (**B**), AD (**D**), AP (**F**), AT (**H**), ES (**J**), ME (**L**) and RI (**N**) events. (**O**) The coefficient profiles of all seven types of AS events. (**P**) The partial likelihood deviance of all seven types of AS events.

**Figure 5:**
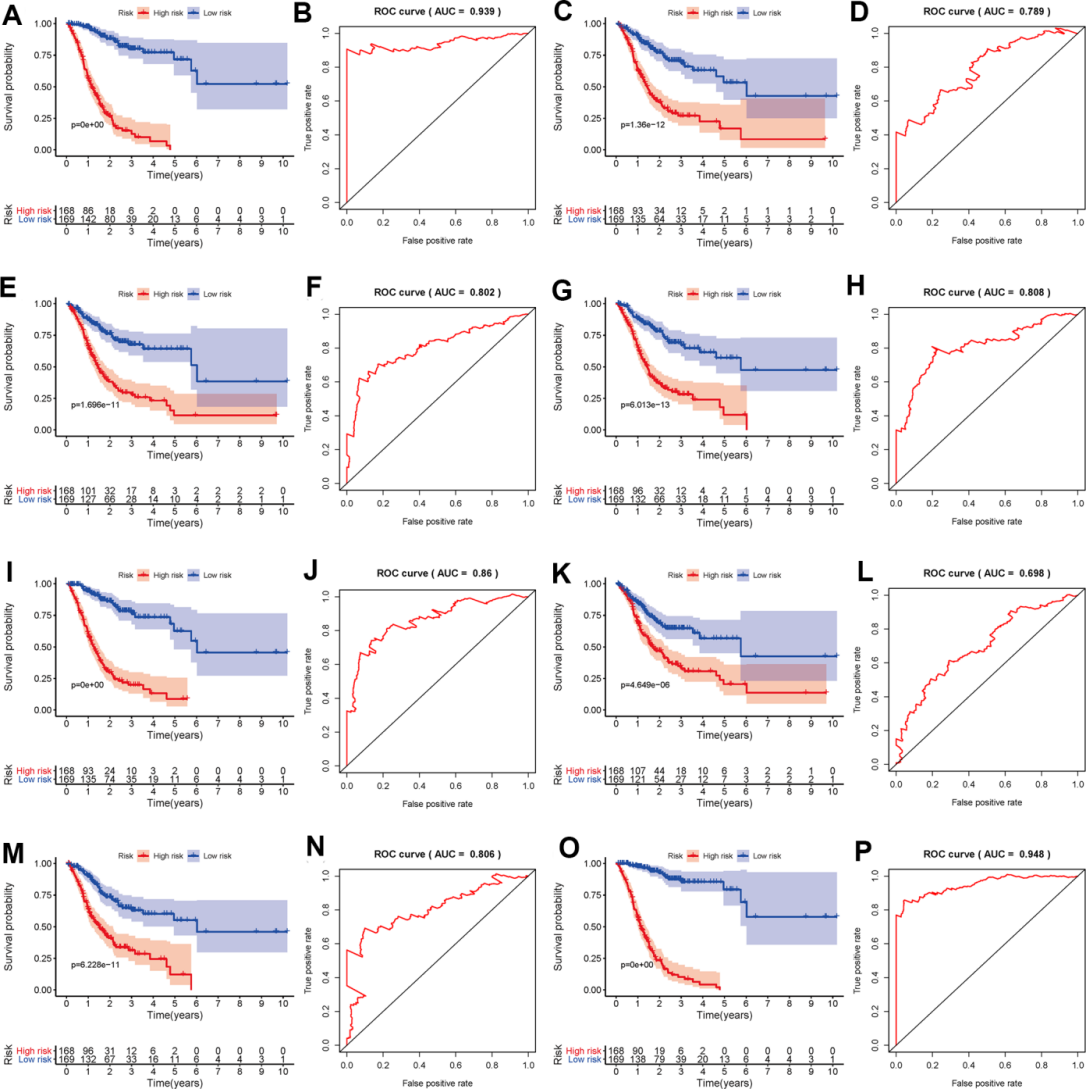
**Kaplan-Meier and ROC curves of prognostic predictors in the GC cohort.** Kaplan-Meier plot of the survival probability over time for prognostic predictors based on AA (**A**), AD (**C**), AP (**E**), AT (**G**), ES (**I**), ME (**K**) and RI (**M**) events with high (red) and low (blue) risk groups, respectively. ROC analysis for prognostic predictors based on AA (**B**), AD (**D**), AP (**F**), AT (**H**), ES (**J**), ME (**L**) and RI (**N**) events. (**O**) Kaplan-Meier plot depicting the survival probability over time for the final prognostic predictor with high (red) and low (blue) risk groups. (**P**) ROC analysis for the final prognostic predictor based on all seven types of AS events.

**Figure 6:**
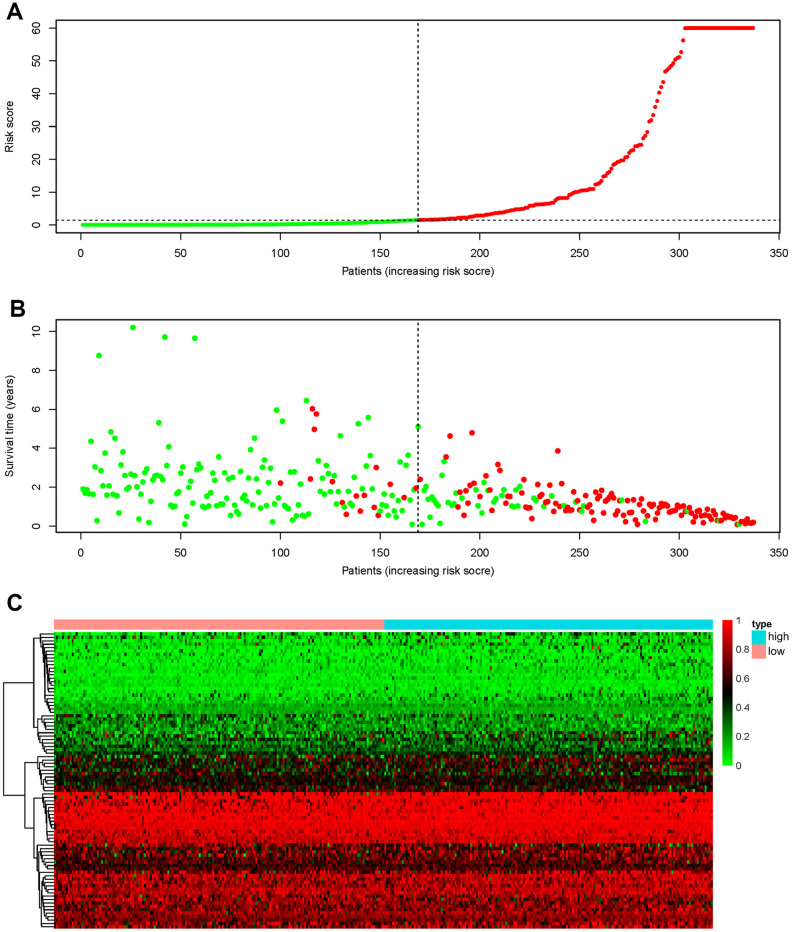
**Recognition capability of the final prognostic signature for GC patients into low- and high-risk groups.** (**A**) The risk scores of 337 patients. Green/red dots represent low/high risk groups that are distinguished using the dotted lines. (**B**) Overall survival status and survival duration of GC patients. Dotted lines were used to distinguish patients in the high- and low-risk groups. Green dots represent surviving patients, while red dots indicate dead patients. (**C**) Heatmap of PSI values of AS events for building the final prognostic signature.

**Figure 7:**
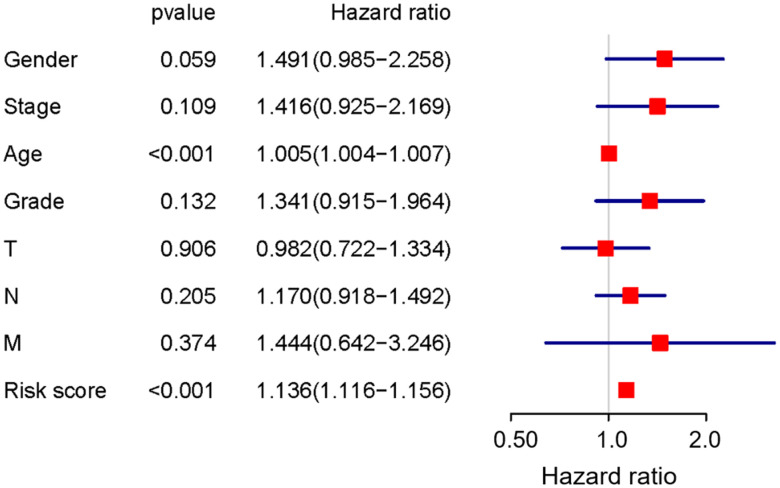
**Prognostic value of the final prognostic signature adjusted by clinical parameters.**

To develop prognostic predictors for GC patient survival, the top significant survival-associated AS events in the seven types were selected as candidates. Least absolute shrinkage and selection operator (LASSO) Cox analysis was carried out to develop seven prognostic signatures based on AA, AD, AP, AT, ES, ME and RI events (Figure 4). As shown in Figure 5, all seven prognostic models built on each type of splicing pattern showed strong potential to predict the outcome of GC patients. Simultaneously, each prognostic model had significant discrepancies for predicting the survival probability, and the AA-based model showed the most promising outcome prediction among the seven prognostic models. The area under the curve (AUC) of the receiver operating characteristic (ROC) for the AA model was 0.939 followed by the ES, AT, RI, AP, AD and ME models with AUCs of 0.860, 0.808, 0.806, 0.802, 0.789 and 0.698, respectively. Furthermore, these prognostic AS events in seven different types were combined to build the final prognostic signature. Notably, the final prognostic predictor indeed showed better performance in predicting the outcome of GC patients with an AUC of 0.948 (Figure 5P). The final prognostic model provided an indicator to predict the prognosis of GC patients (Figure 6A). Kaplan-Meier plots indicated that GC patients in the high-risk group had significantly shorter overall survival than those in the low-risk group (Figure 6B), showing that this signature could effectively distinguish GC patients. The percent spliced in (PSI) values of AS events for building the final prognostic model are shown in Figure 6C. After multivariate adjustment for clinical parameters, the prognostic signature can still act as an independent prognostic indicator (hazard ratio (HR)=1.136, 95% confidence interval (CI): 1.116~1.156, *P*<0.001) (Figure 7).

### Correlation between survival-associated AS events and splicing factor expression

**Figure 8:**
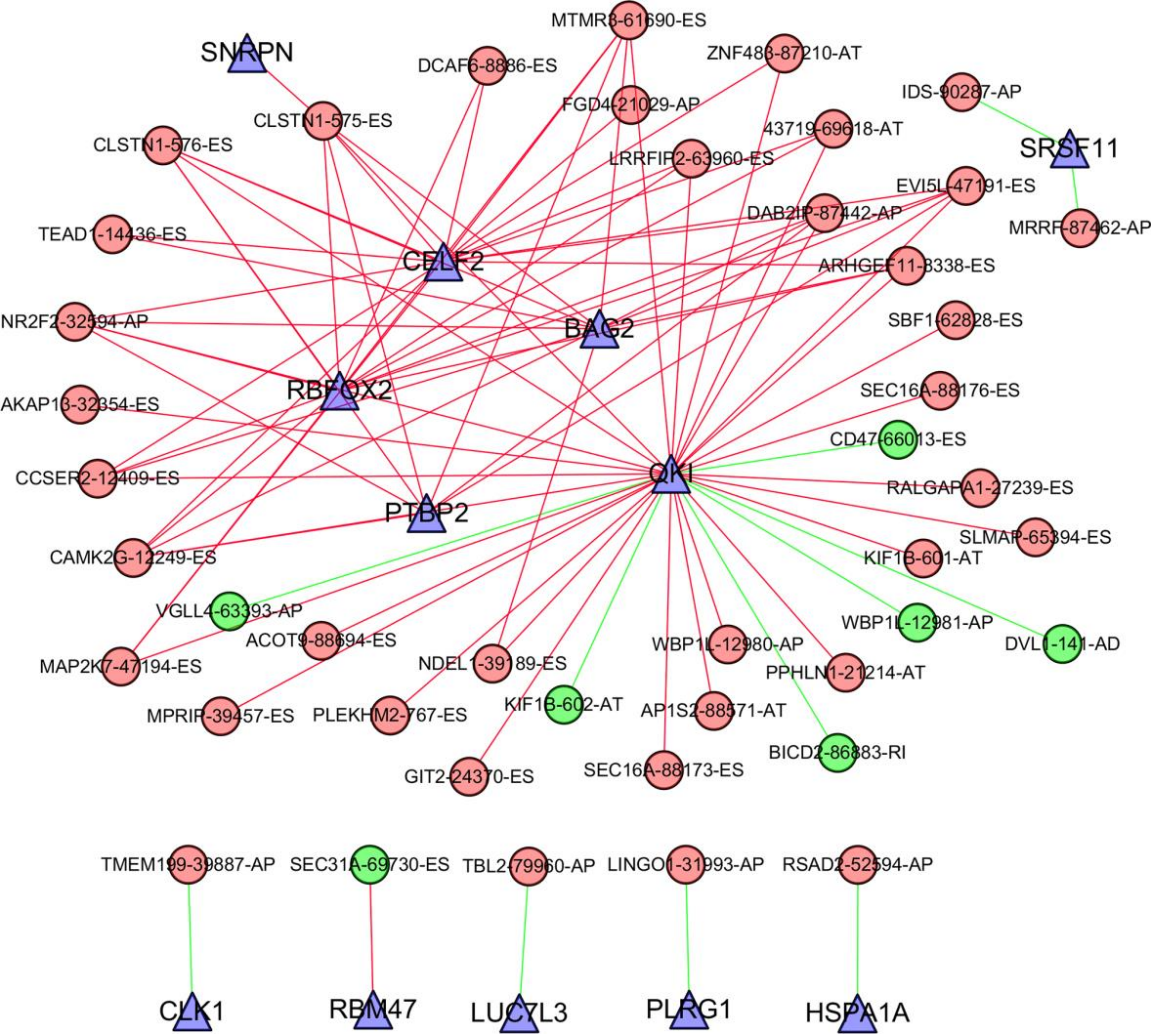
**Correlation network between the expression of splicing factors and PSI values of AS events generated using Cytoscape.** Triangle bubbles represent splicing factors. Red/green round bubbles represent adverse/favourable AS events. Red/green lines represent positive/negative correlations between substances.

AS is mainly orchestrated by splicing factors, which recognize and bind to pre-mRNAs at specific positions and regulate RNA splicing. To explore the correlation between the expression of splicing factors and the PSI values of AS events, Cytoscape software was applied to visualize the splicing-regulatory network of splicing factors and survival-associated AS events. As shown in Figure 8, 12 splicing factors (blue triangles) were significantly associated with 44 survival-associated AS events; among these events, seven were favourable prognosis AS events (green dots), while 37 were poor prognosis AS events (red dots). Moreover, the majority of adverse AS events were positively correlated (red lines) with splicing factor expression, whereas the majority of favourable AS events were negatively correlated (green lines) with splicing factor expression. In addition, five splicing factors, including *CELF2*, *BAG2*, *RBFOX2*, *PTBP2* and *QKI*, were associated with more than two AS events. Among these factors, the expression of *CELF2*, *BAG2*, *RBFOX2* and *PTBP2* was positively correlated with adverse AS events, whereas *QKI* expression was positively correlated with partial adverse AS events and negatively correlated with favourable AS events.

### Functional enrichment analysis

**Figure 9:**
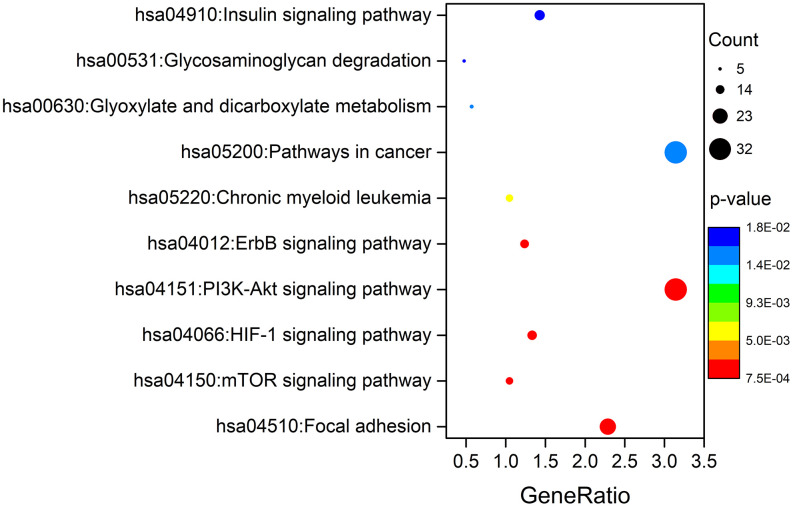
**KEGG pathway analysis of genes with survival-associated AS events.**

To elucidate the function of genes with survival-associated AS events, Gene Ontology (GO) term enrichment and Kyoto Encyclopedia of Genes and Genomes (KEGG) pathway analyses were carried out. As shown in Table 1, “positive regulation of GTPase activity”, “regulation of cell cycle”, and “DNA repair” were the three most significant biological process terms; “protein binding”, “guanyl-nucleotide exchange factor activity” and “microtubule binding” were the three most significant molecular function terms; “cytosol”, “nucleoplasm” and “cytoplasm” were the three most significant cellular component terms. Furthermore, we found that the ten signaling pathways significantly correlated with these genes were focal adhesion, the mTOR signaling pathway, the HIF-1 signaling pathway, the PI3K-Akt signaling pathway, the ErbB signaling pathway, chronic myeloid leukaemia, pathways in cancer, glyoxylate and dicarboxylate metabolism, glycosaminoglycan degradation, and the insulin signaling pathway (Figure 9).

**Table 1:** GO analysis of genes with survival-associated AS events.

**Ontology**	**ID**	**Description**	**p. adjust**	**Count**
Biological process	GO:0043547	positive regulation of GTPase activity	7.66E-04	51
	GO:0051726	regulation of cell cycle	0.001398	17
	GO:0006281	DNA repair	0.001411	26
	GO:0043407	negative regulation of MAP kinase activity	0.003236	8
	GO:0030522	intracellular receptor signaling pathway	0.004442	8
	GO:0043484	regulation of RNA splicing	0.004573	7
	GO:0009612	response to mechanical stimulus	0.004988	10
	GO:0007265	Ras protein signal transduction	0.005109	11
	GO:0007050	cell cycle arrest	0.005168	17
	GO:0006351	DNA-templated transcription	0.005416	135
Molecular function	GO:0005515	protein binding	3.77E-11	587
	GO:0005085	guanyl-nucleotide exchange factor activity	9.02E-05	19
	GO:0008017	microtubule binding	0.001328	24
	GO:0003684	damaged DNA binding	0.002371	11
	GO:0042802	identical protein binding	0.004441	60
	GO:0008307	structural constituent of muscle	0.007928	8
	GO:0005089	Rho guanyl-nucleotide exchange factor activity	0.010129	11
	GO:0070300	phosphatidic acid binding	0.01015	5
	GO:0004672	protein kinase activity	0.010378	32
	GO:0033130	acetylcholine receptor binding	0.011224	4
Cellular component	GO:0005829	cytosol	1.14E-11	261
	GO:0005654	nucleoplasm	8.49E-11	224
	GO:0005737	cytoplasm	8.81E-09	360
	GO:0005634	nucleus	4.99E-05	346
	GO:0005925	focal adhesion	6.65E-05	41
	GO:0016607	nuclear speck	0.001156	23
	GO:0045111	intermediate filament cytoskeleton	0.001594	10
	GO:0005875	microtubule associated complex	0.001853	8
	GO:0035267	NuA4 histone acetyltransferase complex	0.002072	6
	GO:0005874	microtubule	0.002649	30

## DISCUSSION

In 2010, Miura et al. summarized aberrant splicing and altered variant expression in gastrointestinal malignancies and noted the significance of AS in normal and malignant tissues [13]. Growing evidence further demonstrates that aberrant AS events can play an important role in cancer development [6, 14]. At the same time, AS events have shown great prognostic value in various cancer patients. For example, Lin et al. developed a final prognostic signature that can act as an independent prognostic factor for papillary thyroid cancer patients [15]. Lin et al. also highlighted the prognostic value of AS events and splicing factors in gastrointestinal pan-adenocarcinomas [16]. The only analysis of AS events in GC patients has many limitations [12], and it remains urgent to conduct a systematic analysis referring to alterations in the AS patterns and their clinical significance and underlying molecular function, which may help cancer researchers effectively recognize the widespread applicability of AS events in GC patients.

In this study, we downloaded AS profiles from TCGA SpliceSeq and comprehensively investigated the prognostic value of AS events in GC patients. First, we conducted SpliceSeq analyses to generate AS profiles in the GC cohort, which identified 48141 AS events of 10610 genes and verified more than one type of mRNA splicing event in a single gene. Then, a univariate Cox regression analysis was applied to estimate the association of overall survival and AS events; this analysis confirmed the prognostic value of AS events in GC patients. Subsequently, we proposed prognostic predictors for GC patients. All seven prediction models built by individual AS patterns showed considerable potential applications for the prognosis of GC patients. AA events displayed the highest efficiency in distinguishing survival outcome of GC patients, which is similar in colorectal cancer patients [17]. ES events have the highest incidence in GC patients. The AUCs of the ROC curves for the AA and ES models were 0.939 and 0.860, respectively, indicating that these prognostic models might be useful for risk stratification in GC patients. Moreover, a final prognostic predictor model was successfully constructed by the combination of all seven types of AS patterns. The AUC of ROC for this final high-performance model reached 0.948, implying that this model could be more precise in GC prognosis. This model was deployed to recognize GC patients, and the results clearly showed that it could distinguish the GC cohort with distinct clinical outcomes notably well. In addition, the White and Asian GC cohorts were verified to be properly fitted with the prognostic model (Supplementary Figure 2). Thus, the final prognostic signature was an ideal indicator to predict the prognosis of GC patients. Certainly, the present study inevitably had several limitations. First, the number of patients were limited and another independent validation in a larger clinical cohort should be performed in the future study. Second, more in vitro and in vivo experiments are required to elucidate the biological function of these AS events and splicing factors. In addition, this final model may not always effective in the clinical prediction owing to tumor heterogeneity, individual differences and the effects of various treatment options on GC patients.

Generally, the occurrence of AS events is regulated by cis-acting regulatory sequences and RNA-binding protein splicing factors. Splicing factors can act as activators or repressors, depending on their recognition and binding position in a pre-mRNA [1]. Errors in splicing profiles can lead to disease states, which was also verified in multiple tumor types [1, 15–18]. Meanwhile, the importance of splicing factors in cancer was confirmed. Alterations in splicing factors in various cancers are considered independent molecules involved in the prediction of cancer outcome [19–21]. In GC patients, we found that 12 splicing factors were significantly associated with 44 survival-associated AS events. The splicing correlation network clearly showed that *CELF2*, *BAG2*, *RBFOX2*, *PTBP2* and *QKI* were associated with more than two AS events, confirming that limited splicing factors can dominate numerous AS events [1]. According to existing studies, these splicing factors are highly correlated with the development, progression and prognosis of multiple cancers [22–26]. Notably, *CELF2*, *RBFOX2*, *PTBP2* and *QKI* were also involved in the misregulation of AS events in Epstein-Barr virus-associated gastric carcinomas [11]. The network in our analysis showed that the expression of these five splicing factors was positively correlated with adverse AS events or negatively correlated with favourable AS events, indicating a strongly negative impact on the clinical outcome of GC patients. Thus, it may be valuable to study these splicing factors and their relevant survival-associated AS events, which may provide a new perspective in contrast to current approaches and may help to develop novel therapeutic targets for GC patients.

Further functional enrichment analysis showed that the “focal adhesion” pathway was most significantly correlated with these genes with survival-associated AS events. It is well-known that focal adhesions are stable integrin-mediated, cell-substrate adhesion structures that anchor cells via integrin receptors to the extracellular matrix (ECM) and intracellularly connect to actin stress fibres [27, 28]. These structures have been extensively characterized in cultured cells, in which they not only form a structural link between cells and their microenvironment but are also important for cell differentiation, proliferation, adhesion, and migration [28–32]. A key regulator in focal adhesions is the nonreceptor tyrosine kinase focal adhesion kinase (FAK), which triggers focal adhesion signals on cell adhesion to the ECM [28]. Interestingly, FAK is closely related to relevant signaling pathways, including the mTOR signaling pathway, HIF-1 signaling pathway, and PI3K-Akt signaling pathway, which are synergistically involved in tumor migration, invasion, metastasis and recurrence [33, 34]. Thus, AS events occurring in GC patients might impact tumor invasion and metastasis via the focal adhesion pathway.

In summary, the current study established an ideal independent prognostic signature to predict the prognosis of GC patients based on systematic analyses of AS profiles and survival-associated AS events. The AUC of ROC for the final prognostic predictor was 0.948, showing considerable promise for predicting the outcome of GC patients. Further confirmation analysis clearly revealed good performance in distinguishing the GC cohort with distinct risk scores. Simultaneously, the constructed network reveals potential regulatory mechanisms between splicing factors and survival-associated AS events. In addition, GO enrichment and KEGG pathway analyses elucidated that the genes with survival-associated AS events were closely correlated with tumorigenesis and development, especially focal adhesion and its interdependent signaling pathway. These findings may facilitate ongoing efforts to develop novel diagnostic or prognostic biomarkers and exploit therapeutic targets for GC patients.

## MATERIALS AND METHODS

### Data acquisition

TCGA’s SpliceSeq is a computational tool that provides AS profiles on the basis of RNA-seq data [35]. In GC cohort samples, AS events occurring in ≥75% of samples were downloaded from the SpliceSeq database. Clinical information of GC patients was also downloaded and segregated from the TCGA pan-cancer atlas database [36]. AS event data and clinical information were confirmed using the same TCGA ID. The primary tumor characteristics and clinical information were listed in Supplementary Table 2. Subsequent analyses were conducted and the flow chart of data processing are shown in Supplementary Figure 1.

### Identification of survival-associated AS events

In the survival analysis, only GC patients with both clinical follow-up and AS event data were finally enrolled in this study. Simultaneously, the cases in the following two criteria were excluded: (1) the overall survival time of patients less than 30 days; (2) the missing of AS events more than 20%. After AS events with a standard deviation (SD)<0.01 excluded, a univariate Cox analysis was performed to assess the relationships between each AS event and the overall survival of GC patients. UpSet was applied to visualize the associations between genes and each type of AS event. The Reactome was used to plot the protein networks to explore the interactions between the genes corresponding to survival-associated AS events.

### Construction of prognostic predictors

The survival-associated AS events were selected for multivariate LASSO Cox analysis to develop prognostic signatures based on AA, AD, AP, AT, ES, ME and RI events. Subsequently, prognostic models were constructed, and Kaplan-Meier analysis was used to plot the survival probability over time for prognostic predictors of seven types of AS events. Then, the final prognostic model was constructed by the combination of all seven types of AS patterns. At the same time, ROC analyses were performed to assess the validity of prognostic predictors. The prognostic model was constructed to predict the clinical outcomes of GC patients. To further check the validity of the final prognostic model, the Kaplan-Meier method was deployed to plot the relationships between the survival outcome of GC patients with high and low risk scores. Finally, the final model was adjusted using a multivariate Cox regression analysis of sex, stage, age, tumor grade and the risk score.

### Construction of the correlation network of survival-associated AS events and splicing factors

A catalogue of 404 splicing factor genes was referred to a previous study [37]. The expression profiles of splicing factor genes were obtained from the TCGA database. The count value of splicing factor level-3 mRNA data was also downloaded and converted to log_2_(count+1). Cytoscape software version 3.7.1 was used to generate the correlation network between the expression of splicing factors and PSI values of survival-associated AS events. We set the parameters of *P*<0.001 and Pearson correlation coefficient ®>0.6.

### Functional annotation

ClusterProfiler was applied to comprehensively perform GO term enrichment and KEGG analyses of 1088 genes corresponding to the significant survival-associated AS events. We considered the categories with a *P*<0.05 to be significant and displayed the top 10 enrichments of each sub-ontology and pathways.

## Supplementary Material

Supplementary Figures

Supplementary Table 1

Supplementary Table 2
