# Supplementary material for: Prognostic significance of survival-associated alternative splicing events in gastric cancer
Source: Aging (Albany NY). 2020 Nov 7;12(21):21923–41. doi: 10.18632/aging.104013 (PMC7695385; doi:10.18632/aging.104013)
Supplement: Supplementary Figures [file aging-12-104013-s001..pdf]

## SUPPLEMENTARY FIGURES

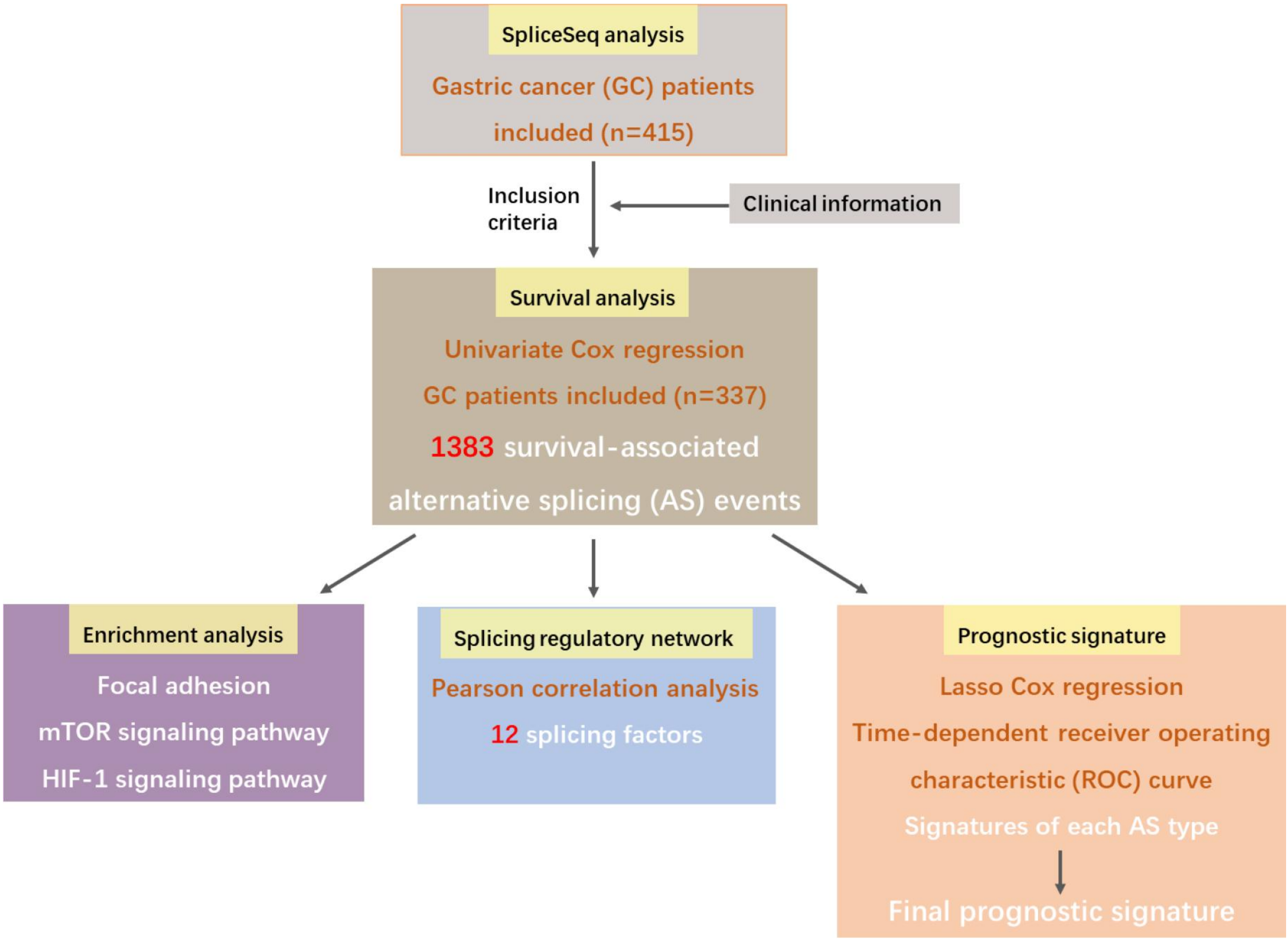


**Supplementary Figure 1. Flow chart of data processing in this study.** In gastric cancer (GC) cohort samples, alternative splicing (AS) events occurring in ≥75% of samples were downloaded from the SpliceSeq database. The patient samples with both clinical follow-up and AS event data were enrolled in our study. In result, we conducted a SpliceSeq analysis based on the 415 GC cases. In the survival analysis, 47 patients with an overall survival time of less than 30 days were excluded. The remaining 368 patients were then matched with their corresponding entries in the SpliceSeq database, and 31 cases were excluded once again due to >20% missing AS events. Thus, 337 patients were included in this study and their overall survival status was listed in Supplementary Table 2. A total of 1383 AS events were found to be significantly associated with the overall survival of GC patients, including 517 exon skip (ES) events, 354 alternate promoter (AP) events, 225 alternate terminator (AT) events, 98 alternate acceptor site (AA) events, 104 alternate donor site (AD) events, 72 retained intron (RI) events, and 13 mutually exclusive exons (ME) events. The functional enrichment analyses were carried out to elucidate the function of genes with survival-associated AS events. Cytoscape software was applied to visualize the splicing-regulatory network of splicing factors and survival-associated AS events to explore the correlation between the expression of splicing factors and the PSI values of AS events. LASSO Cox analysis was carried out to develop seven prognostic signatures based on AA, AD, AP, AT, ES, ME and RI events. Furthermore, these prognostic AS events in seven different types were combined to build the final prognostic signature.

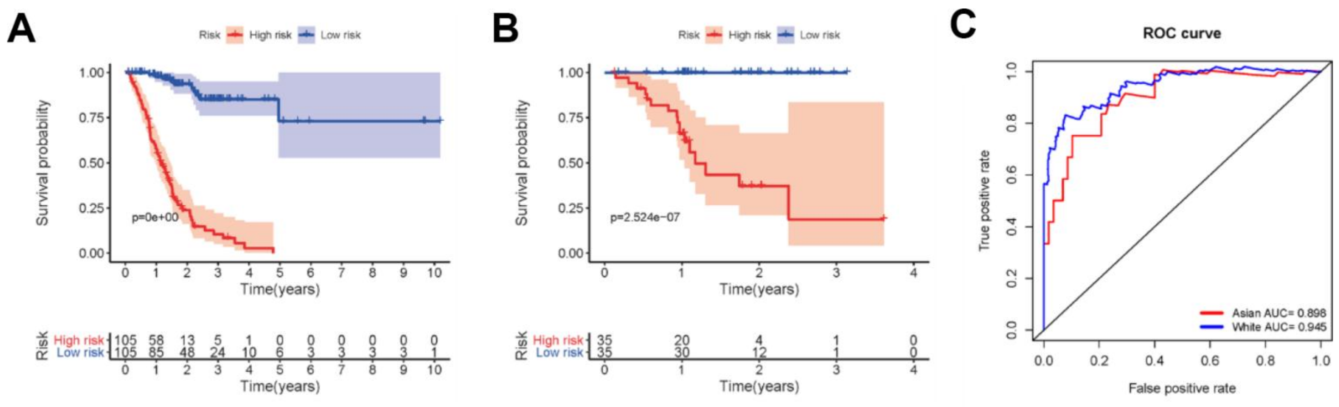


**Supplementary Figure 2. Kaplan-Meier and receiver operating characteristic (ROC) curves fitted with the White and Asian gastric cancer (GC) cohorts.** Kaplan-Meier plot of the survival probability over time for White (**A**) and Asian (**B**) GC cohort with high (red) and low (blue) risk groups, respectively. (**C**) ROC analysis for White (blue) and Asian (red) GC cohort. The significance level of ROC curves between White and Asian cohort was compared and the *p*-value was 0.73.
