# Supplementary material for: Prognostic significance of survival-associated alternative splicing events in gastric cancer
Source: Aging (Albany NY). 2020 Nov 7;12(21):21923–41. doi: 10.18632/aging.104013 (PMC7695385; doi:10.18632/aging.104013)
Supplement: Supplementary Table 1 [file aging-12-104013-s002..docx]

Supplementary table

**Supplementary Table 1.** **The overall survival status of the patients**.

| **No.** | **patient_id** | **last_follow_up(years)** | **status** |
| --- | --- | --- | --- |
| 1 | TCGA_RD_A8MV | 10.19178082 | Alive |
| 2 | TCGA_RD_A8N2 | 9.698630137 | Alive |
| 3 | TCGA_RD_A8N1 | 9.64109589 | Alive |
| 4 | TCGA_CG_5723 | 8.756164384 | Alive |
| 5 | TCGA_HF_7132 | 6.44109589 | Alive |
| 6 | TCGA_F1_6875 | 6.019178082 | Dead |
| 7 | TCGA_RD_A8N4 | 5.947945205 | Alive |
| 8 | TCGA_CG_5732 | 5.753424658 | Dead |
| 9 | TCGA_VQ_A92D | 5.567123288 | Alive |
| 10 | TCGA_VQ_A8PX | 5.380821918 | Alive |
| 11 | TCGA_KB_A6F7 | 5.301369863 | Alive |
| 12 | TCGA_HF_7133 | 5.254794521 | Alive |
| 13 | TCGA_VQ_A91K | 5.101369863 | Alive |
| 14 | TCGA_FP_8209 | 4.961643836 | Dead |
| 15 | TCGA_3M_AB46 | 4.835616438 | Alive |
| 16 | TCGA_RD_A8N5 | 4.78630137 | Dead |
| 17 | TCGA_VQ_A91Z | 4.630136986 | Alive |
| 18 | TCGA_VQ_A924 | 4.619178082 | Dead |
| 19 | TCGA_VQ_AA6F | 4.509589041 | Alive |
| 20 | TCGA_CG_4438 | 4.506849315 | Alive |
| 21 | TCGA_HF_7134 | 4.350684932 | Alive |
| 22 | TCGA_VQ_A8DT | 4.065753425 | Alive |
| 23 | TCGA_CG_4444 | 3.920547945 | Alive |
| 24 | TCGA_VQ_A8PC | 3.854794521 | Dead |
| 25 | TCGA_R5_A7O7 | 3.805479452 | Alive |
| 26 | TCGA_BR_6852 | 3.745205479 | Alive |
| 27 | TCGA_VQ_AA68 | 3.638356164 | Alive |
| 28 | TCGA_VQ_A8E2 | 3.61369863 | Alive |
| 29 | TCGA_VQ_A91V | 3.553424658 | Alive |
| 30 | TCGA_VQ_A94R | 3.545205479 | Dead |
| 31 | TCGA_RD_A8N0 | 3.38630137 | Alive |
| 32 | TCGA_BR_6801 | 3.350684932 | Alive |
| 33 | TCGA_BR_7716 | 3.315068493 | Alive |
| 34 | TCGA_VQ_A91A | 3.287671233 | Alive |
| 35 | TCGA_VQ_A8P2 | 3.178082192 | Alive |
| 36 | TCGA_RD_A8MW | 3.15890411 | Dead |
| 37 | TCGA_KB_A93H | 3.136986301 | Alive |
| 38 | TCGA_VQ_A8E7 | 3.117808219 | Alive |
| 39 | TCGA_BR_8058 | 3.104109589 | Alive |
| 40 | TCGA_VQ_A8P3 | 3.101369863 | Alive |
| 41 | TCGA_KB_A93J | 3.079452055 | Alive |
| 42 | TCGA_D7_A4YX | 3.035616438 | Alive |
| 43 | TCGA_IN_7806 | 3.030136986 | Alive |
| 44 | TCGA_BR_7703 | 3.01369863 | Alive |
| 45 | TCGA_CG_5718 | 3 | Dead |
| 46 | TCGA_BR_7707 | 2.98630137 | Alive |
| 47 | TCGA_RD_A8N9 | 2.967123288 | Alive |
| 48 | TCGA_BR_7704 | 2.936986301 | Alive |
| 49 | TCGA_BR_6452 | 2.890410959 | Alive |
| 50 | TCGA_VQ_A8PB | 2.857534247 | Dead |
| 51 | TCGA_BR_A44T | 2.843835616 | Alive |
| 52 | TCGA_BR_7715 | 2.802739726 | Alive |
| 53 | TCGA_HU_A4HD | 2.783561644 | Alive |
| 54 | TCGA_BR_7959 | 2.767123288 | Alive |
| 55 | TCGA_VQ_A91S | 2.739726027 | Alive |
| 56 | TCGA_BR_6566 | 2.731506849 | Alive |
| 57 | TCGA_BR_8682 | 2.715068493 | Alive |
| 58 | TCGA_BR_A4J7 | 2.709589041 | Alive |
| 59 | TCGA_BR_8081 | 2.687671233 | Alive |
| 60 | TCGA_BR_8680 | 2.663013699 | Alive |
| 61 | TCGA_BR_8372 | 2.605479452 | Alive |
| 62 | TCGA_BR_6803 | 2.6 | Alive |
| 63 | TCGA_BR_8361 | 2.591780822 | Alive |
| 64 | TCGA_VQ_A8P8 | 2.580821918 | Alive |
| 65 | TCGA_CG_4477 | 2.580821918 | Alive |
| 66 | TCGA_BR_4201 | 2.575342466 | Dead |
| 67 | TCGA_BR_6802 | 2.575342466 | Alive |
| 68 | TCGA_HF_A5NB | 2.542465753 | Alive |
| 69 | TCGA_HU_A4GN | 2.498630137 | Alive |
| 70 | TCGA_CG_4443 | 2.498630137 | Alive |
| 71 | TCGA_BR_7958 | 2.463013699 | Alive |
| 72 | TCGA_BR_8286 | 2.452054795 | Alive |
| 73 | TCGA_HU_A4H3 | 2.416438356 | Alive |
| 74 | TCGA_HU_8249 | 2.41369863 | Alive |
| 75 | TCGA_CG_5726 | 2.41369863 | Dead |
| 76 | TCGA_BR_7723 | 2.394520548 | Dead |
| 77 | TCGA_BR_A4IV | 2.380821918 | Dead |
| 78 | TCGA_VQ_AA69 | 2.367123288 | Alive |
| 79 | TCGA_BR_A4J5 | 2.361643836 | Alive |
| 80 | TCGA_BR_8591 | 2.345205479 | Alive |
| 81 | TCGA_VQ_AA6J | 2.295890411 | Alive |
| 82 | TCGA_VQ_A8PU | 2.279452055 | Dead |
| 83 | TCGA_BR_8589 | 2.260273973 | Alive |
| 84 | TCGA_VQ_A94U | 2.243835616 | Alive |
| 85 | TCGA_BR_8677 | 2.22739726 | Alive |
| 86 | TCGA_HJ_7597 | 2.205479452 | Dead |
| 87 | TCGA_BR_8367 | 2.194520548 | Dead |
| 88 | TCGA_BR_6564 | 2.175342466 | Dead |
| 89 | TCGA_HU_A4GF | 2.150684932 | Alive |
| 90 | TCGA_D7_5577 | 2.142465753 | Dead |
| 91 | TCGA_BR_6705 | 2.134246575 | Dead |
| 92 | TCGA_BR_8484 | 2.098630137 | Dead |
| 93 | TCGA_BR_8382 | 2.087671233 | Dead |
| 94 | TCGA_BR_8678 | 2.065753425 | Alive |
| 95 | TCGA_D7_8570 | 2.060273973 | Alive |
| 96 | TCGA_HU_8244 | 2.032876712 | Alive |
| 97 | TCGA_HU_A4G2 | 2.024657534 | Alive |
| 98 | TCGA_HU_A4G9 | 2.016438356 | Alive |
| 99 | TCGA_HU_A4H4 | 1.98630137 | Alive |
| 100 | TCGA_HU_A4H5 | 1.983561644 | Alive |
| 101 | TCGA_VQ_A8PP | 1.950684932 | Dead |
| 102 | TCGA_CG_4475 | 1.915068493 | Alive |
| 103 | TCGA_HU_8604 | 1.901369863 | Alive |
| 104 | TCGA_HU_A4GD | 1.895890411 | Alive |
| 105 | TCGA_HU_A4G8 | 1.890410959 | Alive |
| 106 | TCGA_BR_6563 | 1.860273973 | Alive |
| 107 | TCGA_HU_8602 | 1.860273973 | Alive |
| 108 | TCGA_FP_7998 | 1.857534247 | Alive |
| 109 | TCGA_D7_A6F0 | 1.857534247 | Alive |
| 110 | TCGA_VQ_A8PE | 1.849315068 | Dead |
| 111 | TCGA_BR_8364 | 1.849315068 | Alive |
| 112 | TCGA_CG_4460 | 1.832876712 | Dead |
| 113 | TCGA_BR_7196 | 1.824657534 | Alive |
| 114 | TCGA_VQ_A91E | 1.819178082 | Alive |
| 115 | TCGA_VQ_A8E3 | 1.810958904 | Dead |
| 116 | TCGA_HU_A4GJ | 1.780821918 | Alive |
| 117 | TCGA_F1_A448 | 1.77260274 | Alive |
| 118 | TCGA_HU_A4H6 | 1.764383562 | Alive |
| 119 | TCGA_D7_8578 | 1.761643836 | Alive |
| 120 | TCGA_HU_8608 | 1.756164384 | Alive |
| 121 | TCGA_VQ_A94O | 1.753424658 | Dead |
| 122 | TCGA_D7_8579 | 1.742465753 | Alive |
| 123 | TCGA_BR_8686 | 1.739726027 | Dead |
| 124 | TCGA_VQ_A91Q | 1.734246575 | Dead |
| 125 | TCGA_D7_6519 | 1.712328767 | Alive |
| 126 | TCGA_D7_A6EZ | 1.693150685 | Dead |
| 127 | TCGA_HU_A4GX | 1.687671233 | Alive |
| 128 | TCGA_BR_8486 | 1.684931507 | Alive |
| 129 | TCGA_D7_A74A | 1.663013699 | Alive |
| 130 | TCGA_BR_8291 | 1.663013699 | Dead |
| 131 | TCGA_BR_6707 | 1.657534247 | Alive |
| 132 | TCGA_MX_A5UJ | 1.643835616 | Alive |
| 133 | TCGA_B7_A5TI | 1.630136986 | Alive |
| 134 | TCGA_IN_A6RN | 1.62739726 | Alive |
| 135 | TCGA_FP_7829 | 1.62739726 | Alive |
| 136 | TCGA_D7_8573 | 1.624657534 | Alive |
| 137 | TCGA_BR_6458 | 1.610958904 | Dead |
| 138 | TCGA_SW_A7EA | 1.58630137 | Alive |
| 139 | TCGA_CG_4466 | 1.580821918 | Alive |
| 140 | TCGA_BR_7851 | 1.57260274 | Dead |
| 141 | TCGA_D7_6520 | 1.569863014 | Alive |
| 142 | TCGA_IN_8462 | 1.567123288 | Alive |
| 143 | TCGA_VQ_A91N | 1.561643836 | Dead |
| 144 | TCGA_D7_6522 | 1.550684932 | Alive |
| 145 | TCGA_D7_6521 | 1.545205479 | Alive |
| 146 | TCGA_VQ_A8E0 | 1.539726027 | Dead |
| 147 | TCGA_VQ_AA64 | 1.534246575 | Dead |
| 148 | TCGA_IN_A6RI | 1.531506849 | Alive |
| 149 | TCGA_BR_4191 | 1.528767123 | Dead |
| 150 | TCGA_D7_8575 | 1.517808219 | Dead |
| 151 | TCGA_R5_A7ZE | 1.517808219 | Dead |
| 152 | TCGA_BR_7717 | 1.512328767 | Dead |
| 153 | TCGA_VQ_A8PK | 1.487671233 | Dead |
| 154 | TCGA_D7_6524 | 1.487671233 | Alive |
| 155 | TCGA_BR_8365 | 1.460273973 | Dead |
| 156 | TCGA_BR_6456 | 1.44109589 | Dead |
| 157 | TCGA_ZA_A8F6 | 1.438356164 | Alive |
| 158 | TCGA_D7_8574 | 1.432876712 | Alive |
| 159 | TCGA_D7_6526 | 1.432876712 | Alive |
| 160 | TCGA_VQ_AA6D | 1.42739726 | Alive |
| 161 | TCGA_FP_8099 | 1.421917808 | Alive |
| 162 | TCGA_RD_A8NB | 1.405479452 | Dead |
| 163 | TCGA_D7_8572 | 1.4 | Alive |
| 164 | TCGA_RD_A7C1 | 1.389041096 | Dead |
| 165 | TCGA_D7_A4YU | 1.369863014 | Alive |
| 166 | TCGA_VQ_A8PD | 1.35890411 | Dead |
| 167 | TCGA_BR_A4QL | 1.345205479 | Dead |
| 168 | TCGA_D7_6815 | 1.331506849 | Alive |
| 169 | TCGA_CG_4305 | 1.328767123 | Alive |
| 170 | TCGA_BR_6453 | 1.328767123 | Alive |
| 171 | TCGA_IN_AB1V | 1.312328767 | Alive |
| 172 | TCGA_HU_A4HB | 1.306849315 | Dead |
| 173 | TCGA_VQ_A8PQ | 1.304109589 | Dead |
| 174 | TCGA_D7_A6F2 | 1.304109589 | Alive |
| 175 | TCGA_BR_8296 | 1.298630137 | Dead |
| 176 | TCGA_CD_8533 | 1.282191781 | Alive |
| 177 | TCGA_BR_7722 | 1.276712329 | Dead |
| 178 | TCGA_D7_6528 | 1.268493151 | Alive |
| 179 | TCGA_CG_5725 | 1.252054795 | Dead |
| 180 | TCGA_BR_8373 | 1.232876712 | Alive |
| 181 | TCGA_D7_A4Z0 | 1.230136986 | Alive |
| 182 | TCGA_D7_8576 | 1.221917808 | Dead |
| 183 | TCGA_F1_6874 | 1.205479452 | Alive |
| 184 | TCGA_BR_8059 | 1.202739726 | Dead |
| 185 | TCGA_FP_7916 | 1.17260274 | Dead |
| 186 | TCGA_HU_A4H8 | 1.17260274 | Alive |
| 187 | TCGA_MX_A666 | 1.169863014 | Alive |
| 188 | TCGA_BR_8369 | 1.169863014 | Alive |
| 189 | TCGA_CG_4441 | 1.167123288 | Dead |
| 190 | TCGA_BR_A44U | 1.156164384 | Dead |
| 191 | TCGA_BR_6455 | 1.156164384 | Dead |
| 192 | TCGA_BR_6457 | 1.139726027 | Alive |
| 193 | TCGA_FP_8211 | 1.131506849 | Alive |
| 194 | TCGA_BR_A4J8 | 1.126027397 | Alive |
| 195 | TCGA_IN_AB1X | 1.126027397 | Alive |
| 196 | TCGA_CD_5798 | 1.117808219 | Alive |
| 197 | TCGA_D7_6525 | 1.112328767 | Dead |
| 198 | TCGA_IN_A6RL | 1.112328767 | Dead |
| 199 | TCGA_VQ_A8DV | 1.104109589 | Dead |
| 200 | TCGA_CD_5801 | 1.098630137 | Dead |
| 201 | TCGA_CD_5800 | 1.095890411 | Alive |
| 202 | TCGA_VQ_A8DZ | 1.084931507 | Dead |
| 203 | TCGA_CD_5799 | 1.084931507 | Alive |
| 204 | TCGA_HU_A4H2 | 1.079452055 | Alive |
| 205 | TCGA_CD_8535 | 1.068493151 | Alive |
| 206 | TCGA_VQ_A8PH | 1.065753425 | Dead |
| 207 | TCGA_BR_8588 | 1.065753425 | Alive |
| 208 | TCGA_CD_8524 | 1.063013699 | Alive |
| 209 | TCGA_D7_5578 | 1.054794521 | Alive |
| 210 | TCGA_CD_8525 | 1.049315068 | Alive |
| 211 | TCGA_CD_8531 | 1.049315068 | Alive |
| 212 | TCGA_IN_A6RS | 1.049315068 | Alive |
| 213 | TCGA_CD_8526 | 1.043835616 | Alive |
| 214 | TCGA_IN_A6RJ | 1.038356164 | Alive |
| 215 | TCGA_VQ_AA6K | 1.035616438 | Dead |
| 216 | TCGA_CD_A48A | 1.035616438 | Alive |
| 217 | TCGA_CD_5813 | 1.032876712 | Dead |
| 218 | TCGA_CD_8530 | 1.032876712 | Alive |
| 219 | TCGA_D7_6818 | 1.030136986 | Dead |
| 220 | TCGA_CD_8528 | 1.02739726 | Alive |
| 221 | TCGA_D7_6822 | 1.02739726 | Alive |
| 222 | TCGA_CD_8529 | 1.024657534 | Alive |
| 223 | TCGA_CD_A487 | 1.024657534 | Alive |
| 224 | TCGA_CD_A4MH | 1.016438356 | Alive |
| 225 | TCGA_BR_6709 | 1.01369863 | Dead |
| 226 | TCGA_CD_5804 | 1.008219178 | Alive |
| 227 | TCGA_CD_8534 | 1.005479452 | Alive |
| 228 | TCGA_CG_5724 | 1.002739726 | Dead |
| 229 | TCGA_BR_8371 | 0.983561644 | Dead |
| 230 | TCGA_HU_A4GH | 0.980821918 | Alive |
| 231 | TCGA_IN_A7NU | 0.975342466 | Alive |
| 232 | TCGA_VQ_A91D | 0.975342466 | Dead |
| 233 | TCGA_B7_5818 | 0.975342466 | Alive |
| 234 | TCGA_CD_8532 | 0.969863014 | Dead |
| 235 | TCGA_CD_A48C | 0.967123288 | Dead |
| 236 | TCGA_D7_A6EY | 0.953424658 | Dead |
| 237 | TCGA_BR_8060 | 0.953424658 | Dead |
| 238 | TCGA_F1_A72C | 0.947945205 | Alive |
| 239 | TCGA_CD_A489 | 0.942465753 | Dead |
| 240 | TCGA_VQ_A94T | 0.936986301 | Dead |
| 241 | TCGA_D7_A6EV | 0.936986301 | Alive |
| 242 | TCGA_CD_5803 | 0.934246575 | Dead |
| 243 | TCGA_RD_A7BS | 0.920547945 | Dead |
| 244 | TCGA_B7_A5TJ | 0.917808219 | Alive |
| 245 | TCGA_IN_A7NT | 0.884931507 | Alive |
| 246 | TCGA_D7_6527 | 0.854794521 | Dead |
| 247 | TCGA_BR_8683 | 0.821917808 | Dead |
| 248 | TCGA_MX_A663 | 0.821917808 | Dead |
| 249 | TCGA_VQ_A91Y | 0.810958904 | Dead |
| 250 | TCGA_BR_4257 | 0.805479452 | Dead |
| 251 | TCGA_BR_8080 | 0.8 | Dead |
| 252 | TCGA_BR_4279 | 0.797260274 | Dead |
| 253 | TCGA_VQ_A91X | 0.791780822 | Dead |
| 254 | TCGA_B7_A5TK | 0.789041096 | Alive |
| 255 | TCGA_B7_A5TN | 0.78630137 | Alive |
| 256 | TCGA_BR_8590 | 0.778082192 | Dead |
| 257 | TCGA_BR_4256 | 0.778082192 | Dead |
| 258 | TCGA_VQ_A8PO | 0.77260274 | Dead |
| 259 | TCGA_R5_A805 | 0.769863014 | Dead |
| 260 | TCGA_BR_8485 | 0.767123288 | Alive |
| 261 | TCGA_BR_7197 | 0.767123288 | Alive |
| 262 | TCGA_BR_6565 | 0.764383562 | Dead |
| 263 | TCGA_BR_7957 | 0.756164384 | Dead |
| 264 | TCGA_VQ_A922 | 0.753424658 | Dead |
| 265 | TCGA_CG_4465 | 0.750684932 | Dead |
| 266 | TCGA_HU_A4GP | 0.747945205 | Alive |
| 267 | TCGA_BR_6710 | 0.747945205 | Alive |
| 268 | TCGA_RD_A8N6 | 0.745205479 | Dead |
| 269 | TCGA_RD_A7BT | 0.717808219 | Dead |
| 270 | TCGA_R5_A7ZF | 0.709589041 | Dead |
| 271 | TCGA_D7_A747 | 0.698630137 | Dead |
| 272 | TCGA_BR_8687 | 0.684931507 | Dead |
| 273 | TCGA_BR_8284 | 0.671232877 | Dead |
| 274 | TCGA_CG_4437 | 0.671232877 | Alive |
| 275 | TCGA_EQ_8122 | 0.665753425 | Dead |
| 276 | TCGA_CG_5734 | 0.665753425 | Dead |
| 277 | TCGA_CG_4436 | 0.665753425 | Alive |
| 278 | TCGA_VQ_A8P5 | 0.643835616 | Dead |
| 279 | TCGA_BR_8297 | 0.616438356 | Alive |
| 280 | TCGA_BR_8381 | 0.61369863 | Alive |
| 281 | TCGA_CD_8527 | 0.597260274 | Dead |
| 282 | TCGA_CG_4469 | 0.589041096 | Dead |
| 283 | TCGA_CG_5717 | 0.580821918 | Dead |
| 284 | TCGA_IN_A6RR | 0.561643836 | Dead |
| 285 | TCGA_BR_4280 | 0.550684932 | Dead |
| 286 | TCGA_F1_6177 | 0.547945205 | Dead |
| 287 | TCGA_VQ_A927 | 0.547945205 | Dead |
| 288 | TCGA_HU_A4GU | 0.547945205 | Alive |
| 289 | TCGA_CD_A4MG | 0.547945205 | Dead |
| 290 | TCGA_IN_A7NR | 0.542465753 | Alive |
| 291 | TCGA_HU_A4GT | 0.542465753 | Alive |
| 292 | TCGA_CD_A486 | 0.526027397 | Dead |
| 293 | TCGA_BR_8592 | 0.523287671 | Dead |
| 294 | TCGA_FP_A9TM | 0.517808219 | Alive |
| 295 | TCGA_BR_4267 | 0.515068493 | Dead |
| 296 | TCGA_R5_A7ZR | 0.506849315 | Dead |
| 297 | TCGA_CG_5721 | 0.501369863 | Alive |
| 298 | TCGA_SW_A7EB | 0.482191781 | Alive |
| 299 | TCGA_VQ_A928 | 0.476712329 | Dead |
| 300 | TCGA_HU_A4G3 | 0.465753425 | Alive |
| 301 | TCGA_FP_A4BF | 0.460273973 | Dead |
| 302 | TCGA_VQ_A8DU | 0.454794521 | Dead |
| 303 | TCGA_BR_8483 | 0.449315068 | Alive |
| 304 | TCGA_RD_A7BW | 0.42739726 | Dead |
| 305 | TCGA_FP_8210 | 0.419178082 | Dead |
| 306 | TCGA_BR_4187 | 0.38630137 | Dead |
| 307 | TCGA_VQ_A925 | 0.378082192 | Dead |
| 308 | TCGA_D7_A748 | 0.361643836 | Dead |
| 309 | TCGA_BR_8368 | 0.35890411 | Alive |
| 310 | TCGA_BR_4253 | 0.339726027 | Dead |
| 311 | TCGA_CG_4440 | 0.334246575 | Dead |
| 312 | TCGA_BR_8384 | 0.309589041 | Alive |
| 313 | TCGA_MX_A5UG | 0.309589041 | Dead |
| 314 | TCGA_FP_7735 | 0.290410959 | Dead |
| 315 | TCGA_BR_7901 | 0.287671233 | Dead |
| 316 | TCGA_IN_8663 | 0.282191781 | Dead |
| 317 | TCGA_HU_A4GC | 0.271232877 | Alive |
| 318 | TCGA_CG_4301 | 0.252054795 | Alive |
| 319 | TCGA_D7_A6EX | 0.235616438 | Alive |
| 320 | TCGA_VQ_A8PJ | 0.224657534 | Dead |
| 321 | TCGA_VQ_A94P | 0.221917808 | Dead |
| 322 | TCGA_BR_8289 | 0.221917808 | Dead |
| 323 | TCGA_IP_7968 | 0.210958904 | Alive |
| 324 | TCGA_VQ_A8PF | 0.208219178 | Dead |
| 325 | TCGA_BR_8295 | 0.183561644 | Dead |
| 326 | TCGA_HU_A4H0 | 0.175342466 | Alive |
| 327 | TCGA_VQ_A923 | 0.167123288 | Dead |
| 328 | TCGA_VQ_A8PM | 0.156164384 | Dead |
| 329 | TCGA_VQ_A91U | 0.142465753 | Dead |
| 330 | TCGA_HU_8238 | 0.126027397 | Alive |
| 331 | TCGA_BR_A4CS | 0.123287671 | Dead |
| 332 | TCGA_BR_A4PF | 0.095890411 | Alive |
| 333 | TCGA_BR_8487 | 0.093150685 | Alive |
| 334 | TCGA_CG_4306 | 0.084931507 | Dead |
| 335 | TCGA_CG_5719 | 0.084931507 | Alive |
| 336 | TCGA_CG_5722 | 0.082191781 | Alive |
| 337 | TCGA_CG_5720 | 0.082191781 | Dead |
