# Supplementary material for: Prognostic significance of survival-associated alternative splicing events in gastric cancer
Source: Aging (Albany NY). 2020 Nov 7;12(21):21923–41. doi: 10.18632/aging.104013 (PMC7695385; doi:10.18632/aging.104013)
Supplement: Supplementary Table 2 [file aging-12-104013-s003..pdf]

SUPPLEMENTARY TABLE

Supplementary Table 2. Primary tumor characteristics and clinical information.

Variable	Number of samples	Ratio (%)	Valid (%)
Age at diagnosis, y			
≤50	27	8.01	8.11
>50	306	90.80	91.89
Missing	4	1.19	
Tumor type			
Signet Ring Type	9	2.67	5.70
Diffuse Type	58	17.21	36.71
Tubular Type	66	19.58	41.77
Mucinous Type	19	5.64	12.02
Papillary Type	6	1.78	3.80
Not Otherwise Specified (NOS)	179	53.12	
Tumor grade			
1	8	2.37	2.45
2	122	36.20	37.31
3	197	58.46	60.24
Missing	10	2.97	
Gender			
Male	218	64.69	64.88
Female	118	35.01	35.12
Missing	1	0.30	
Race			
White	210	62.32	71.92
Asian	70	20.77	23.97
African American	11	3.26	3.77
Native Hawaiian or other pacific islander	1	0.30	0.34
Missing	45	13.35	
T-stage			
T1	18	5.34	5.42
T2	74	21.96	22.29
T3	154	45.70	46.39
T4	86	25.52	25.90
Missing	5	1.48	
N-stage			
N0	99	29.38	30.46
N1	93	27.60	28.62
N2	69	20.47	21.23

N3	64	18.99	19.69
Missing	12	3.56	
M-stage			
M0	302	89.62	93.50
M1	21	6.23	6.50
Missing	14	4.15	
Stage			
I	47	13.95	14.60
II	106	31.45	32.92
III	135	40.06	41.92
IV	34	10.09	10.56
Missing	15	4.45	
Antireflux treatment			
Yes	33	9.79	20.62
No	127	37.69	79.38
Missing	177	52.52	
Family history of stomach cancer			
Yes	15	4.45	5.62
No	252	74.78	94.38
Missing	70	20.77	
Radiation treatment adjuvant			
Yes	41	12.17	22.65
No	140	41.54	77.35
Missing	156	46.29	
Targeted molecular therapy			
Yes	87	25.82	47.54
No	96	28.48	52.46
Missing	154	45.70	
